# Philip Arestis, Peter Mooslechner and Karin Wagner: Housing market challenges in Europe and the United States: Palgrave Macmillan: Basingstoke, 2010, 269 pp, ISBN 978-0-230-22903-7 (hardback)

**DOI:** 10.1007/s10901-012-9284-7

**Published:** 2012-04-03

**Authors:** Peter Boelhouwer

**Affiliations:** Delft University of Technology, Delft, The Netherlands

This edited volume, containing 11 chapters, is based on papers presented at a conference on “Housing Market Challenges in Europe and the United States—Any solutions available?”, held in September 2008 and organised by the Oesterreichische National Bank (the central bank of Austria). In total, 16 authors from seven countries were involved in this project.

The first chapter serves as the introduction, which is actually little more than a brief statement on the origin of the papers, noting that they were revised in 2010 to account for more recent developments. After this very short statement, the contents of the book and the main conclusions of the 11 chapters are presented. Unfortunately, this introduction offers neither a general theoretical framework nor any reflections by the editors.

In the second chapter, “Housing markets in Europe and in the USA: what are the relevant issues today?”, Peter Mooslechner and Karin Wagner identify some key aspects of the economic relevance of housing markets and analyse these in the context of the credit crunch at the beginning of 2010. The key aspects include the relationship of housing markets to their national but also their international macro-economic surroundings, some fiscal and regulatory issues, and the possibility for central banks to react proactively to asset price movement. On the basis of their analysis the authors conclude that there is no definite answer yet to the question of how central banks should best deal with asset prices. Of course, this is a somewhat disappointing outcome. The good news is that a lot of important work remains to be done by housing market researchers and policy advisors.

The third chapter, on the “Subprime mortgage market and current financial crisis”, by Philip Arestis and Elias Karakitos, sheds light on financial liberalisation and innovations like the subprime mortgage market, which helped create the climate for the financial crisis of August 2007. The authors also discuss recent economic policies to deal with the current financial crisis. Their most important piece of advice is that governments should regulate their financial engineering much more rigorously to prevent too much risk-taking. It was not the financial instruments, like subprime mortgages, themselves but the fraud surrounding them which caused the current economic problems. So, in the view of the authors, it was financial engineering that allowed the US housing bubble to form. A striking standpoint, in the light of the current government cuts in Europe, is their plea for public spending as the most effective way of getting out of the current financial and economic troubles.

In chapter four, Elisabeth Springler and Karin Wagner turn their attention to homeownership in an attempt to identify the “Determinants of homeownership rates: housing finance and the role of the state”. More precisely, they advance a time series model of mortgage debt and the cost of financing an owner-occupied home and apply it to the European and US economies. This model confirms the importance of housing costs for the development of homeownership rates. The results also underscore the importance of the role of the state and highlight the immediate effects that housing policies can have.

Chapter five, by Dieter Gstach, elaborates on the rental market. It contains an interesting review of the literature on the macro-economic role of housing, emphasising its contribution to an understanding of rental housing. The chapter also presents some relevant statistics based on the EU SILC data. Gstach brings a largely neglected option to the fore: that the rental sector could provide an important feedback route for changing housing market conditions.

Chapter six, on “Housing markets, business cycles and economic policies”, by Christophe André and Nathalie Girouard, brings us back to the main theme of this book: the relationship between housing markets and the general economy. The advice offered by these authors has already been articulated by Arestis and Karakitsos in chapter three: restoring conditions for a sustainable development of housing markets will imply reinforcing the supervision and regulation of the financial system, in particular, by controlling the level of leverage, avoiding pro-cyclical provisioning and capital standards. Some other important requirements, in the view of the authors, are risk management and transparency as well as ensuring appropriate underwriting standards. To solve the current problems in our housing markets, André and Girouard are in favour of supporting overall demand through targeted fiscal subsides and are also in favour of restoring the normal functioning of financial markets through the provision of state guarantees.

In chapter seven, we turn back to the rental markets. Montserrat Pareja-Eastaway and Maria Teresa Sánchez-Martines inquire into the development of the Spanish rental sector: “European rental markets: regulation or liberalization? The Spanish case”. They start with an overview of the diverse housing contexts and trends in Europe that have developed over the past few decades. They then describe the main features of their theoretical analysis of the role of the tenure system in providing proper housing. The ensuing Spanish case amounts to a historical overview of failed policy approaches for the rented sector. In their conclusion the authors recommend that the government show positive discrimination towards the rented sector in order to avoid a lack of confidence among tenants and landlords, such as that prevailing in most European countries in the past.

In chapter eight, Guido Wolswijk addresses the fiscal aspects of housing in Europe. By describing the fiscal policy instruments applied in discrete housing markets, his contribution stresses that huge differences still exist between the housing systems in Europe. And because of the differences in the tax treatment of housing in the euro area, there is no single prescription for all countries.

In chapter nine, “Towards a new housing system in transitional countries: the case of Hungary”, József Hegedüs considers the dissolution of the East European housing regimes. He argues that countries in transition followed the same trends because they were facing the same housing market challenges after the transition. Since then, differences between the institutional solutions in housing finance and housing welfare schemes have become more and more pronounced. He also notes that the economic crisis had a big impact on the housing markets in the transitional countries, leading to declining house prices, increasing mortgage arrears, and difficult access to mortgages.

The last three chapters are more instrumental in nature and stray a little from the central theme of the book. In chapter ten, house prices and other housing market data are described from a user perspective by Antony Murphy. Then, in chapter 11, Martin Eiglsperger presents the residential property price indices for the euro area and the EU member states, as compiled by the ECB in co-operation with the EU national central banks. Finally, in chapter 12, Pirmin Fessler, Peter Mooslechner, Martin Schürz and Karin Wagner consider micro-economic data in “Housing and financial wealth in Austria: what can survey data tell us for the analysis of financial stability issues?”.

The overarching objective of this book—namely, to develop more insight in the housing market challenges in Europe and the US under the current economic and financial crisis—is in my opinion well addressed. The chapters form a collection of interesting and highly relevant papers. Recent statistics and developments are combined with excellent, mostly econometric, analyses and empirical work, while highly relevant policy conclusions are drawn. Some of the chapters also contain elaborate literature reviews, which could make this volume useful as a textbook. Unfortunately, the editors do not offer much in the way of a general introduction and have omitted a concluding chapter. Given that the individual papers are well connected to the theme—most of them address the background of the housing crisis and discuss relevant policy solutions—this would not have been a difficult task for the editors to fulfil.

